# ncRNA-Mediated High Expression of LPCAT1 Correlates with Poor Prognosis and Tumor Immune Infiltration of Liver Hepatocellular Carcinoma

**DOI:** 10.1155/2022/1584397

**Published:** 2022-05-16

**Authors:** Qiu Sun, Xudong Liu, Qunlong Peng, Lei Hu, Xiaochun Jiang

**Affiliations:** ^1^Heilongjiang University of Chinese Medicine, Harbin, Heilongjiang Province, China; ^2^College of Pharmacy, Xiangnan University, Chenzhou, China; ^3^Anhui Medical University, Hefei, China

## Abstract

**Purpose:**

To investigate the expression of LPCAT1 in liver hepatocellular carcinoma (LIHC) and its relationship with prognosis and immune infiltration and predict its upstream nonencoding RNAs (ncRNAs).

**Method:**

In this study, expression analysis and survival analysis for LPCAT1 in pan cancers were first performed by using The Cancer Genome Atlas (TCGA) data, which suggested that LPCAT1 might be a potential LIHC oncogene. Then, ncRNAs contributing to the overexpression of LPCAT1 were explored in starBase by a combination of expression analysis, correlation analysis, and survival analysis. Immune cell infiltration of LPCAT1 in LIHC was finally investigated via Tumor Immune Estimation Resource (TIMER).

**Result:**

SNHG3 was observed to be the most promising upstream lncRNA for the hsa-miR-139-5p/LPCAT1 axis in LIHC. In addition, the LPCAT1 level was significantly positively associated with tumor immune cell infiltration, biomarkers of immune cells, and immune checkpoint expression in LIHC.

**Conclusion:**

To summarize, the upregulation of LPCAT1 mediated by ncRNAs is associated with poor prognosis, immune infiltration, and immune checkpoint expression in LIHC.

## 1. Introduction

Liver cancer is a leading cause of cancer-related death, which is the sixth most common cancer type and the fourth most deadly cause of cancer worldwide [1, 2]. Among them, liver hepatocellular carcinoma (LIHC) is the major type of liver cancer, accounting for 80–90% of all cases of primary liver cancer [3]. It exists many causes of LIHC, mainly related to some factors of chronic hepatitis and chronic liver diseases [3, 4]. The most common etiology of liver cancer in our country is hepatitis B, and the second is hepatitis C and then followed by eating habits, living habits, environment, and so on [3–5]. Most of them have varying degrees of cirrhosis. All these risk factors may result in activation of oncogenes and inactivation of tumor suppressor genes and could cause unrestricted growth of LIHC cells [3–5]. The prognosis of advanced liver cancer is still poor despite the many treatments especially molecular target agents and immune checkpoint inhibitors that have been developed in the past few decades. Thus, there are urgent calls for effective methods to diagnose LIHC.

Lysophosphatidylcholine acyltransferases (LPCATs) are enzymes catalyzing lysophosphatidylcholine (LPC) to phosphatidylcholine (PC) conversion [6–8]. Previous studies have reported that PC metabolic dysregulation and subsequent membrane composition alterations were observed in various cancers. Other than that, the expression of LPCAT1 was also obviously increased in various tumors [9]. Emerging evidence showed that upregulation of LPCAT1 promotes cancer cell proliferation and metastasis while knocking down LPCAT1 could inhibit the growth of cancer cells by inducing cell cycle arrest at G0/G1 phase [10]. And on that basis, we further investigated the role of LPCAT1 in LIHC progression.

In this research, expression analysis and survival analysis for LPCAT1 in various human cancers were firstly conducted. Then, the noncoding RNA- (ncRNA-) associated regulation of LPCAT1, involving microRNAs (miRNAs) and long noncoding RNAs (lncRNAs), was explored for LIHC. Lastly, the correlations of the LPCAT1 expression with immune cell infiltration, biomarkers of immune cells, and immune checkpoints in LIHC were also explored. As a result, our findings suggest that ncRNA-mediated upregulation of LPCAT1 correlates with poor outcomes and tumor immune infiltration in HCC patients.

## 2. Materials and Methods

### 2.1. Download, Process, and Analysis of the Cancer Genome Atlas (TCGA) Data

The RNA⁃seq (HTSeq⁃FPKM) data of the eighteen cancers (BLCA, BRCA, CHOL, COAD, ESCA, GBM, HNSC, KICH, KIRC, KIRP, LIHC, LUAD, LUSC, PRAD, READ, STAD, THCA, and UCEC) were derived from the TCGA database (https://genome-cancer.ucsc.edu/).Thegene expression data were normalized and log2 transformed for subsequent analysis. *R* package limma was used for differential expression analysis to observe whether LPCAT1 was differentially expressed in tumor and nontumor samples [11].

### 2.2. Analysis of LPCAT1 Expression and Prognosis in Pan Cancer by the GEPIA Database

The GEPIA database (http://gepia.cancer-pku.cn/) is a web server for gene expression and interactive analysis of cancer and normal tissues, which collects RNA-seq data from 9736 tumor samples and 8587 normal control samples in the TCGA and The Genotype-Tissue Expression (GTEx) datasets [12]. In this research, the GEPIA database was utilized to analyze the LPCAT1 expression in tumor and normal samples and prognosis in pan cancers.

### 2.3. Prediction and Analysis of Upstream miRNAs of LPCAT1

StarBase is a website containing miRNA-related information that can be used to query upstream miRNAs of LPCAT1. Target gene prediction programs (including TargetScan, miRmap, miRanda, PicTar, RNA22, PITA, and microT) in the “miRNA-mRNA” option from the miRNA-Target module were used to search for the miRNA. If the predicted miRNA can be present in more than one procedure, then it will be incorporated in the subsequent analysis. These predicted miRNAs were regarded as candidate miRNAs of LPCAT1, and the miRNA-LPCAT1 regulatory network was then visualized by the Cytoscape software. The miRNAs were screened, and the one with the most significant negative correlation with LPCAT1 in LIHC was selected. Moreover, expression analysis and prognostic analysis were conducted for this miRNA [13].

### 2.4. Prediction and Analysis of Upstream lncRNAs of miRNA

The “miRNA-lncRNA” option from the miRNA-target module in starBase was selected for candidate lncRNAs [13]. The miRNAs selected in the prior step were entered into the search box. It is known that miRNAs can posttranscriptionally regulate the gene expression by binding to mRNAs, inhibiting their translation or causing mRNA degradation, resulting in gene silencing and thus function of gene expression. The competing endogenous RNA (ceRNA) hypothesis revealed that ceRNAs can regulate the gene expression by competitively binding miRNAs. Therefore, the lncRNA should meet the following conditions: (1) negatively correlated with miRNA and (2) positively correlated with mRNA [14–16].

### 2.5. Analysis of Immune Infiltration in LIHC

The TIMER database (https://cistrome.shinyapps.io/timer/) is a database developed specifically for the analysis of immune cell infiltration in a wide range of cancers. Statistical methods confirmed by pathological examination were used to estimate the infiltration of neutrophils, macrophages, dendritic cells, B cells, and CD4+/CD8+ T cells in tumor tissues [17]. In this study, the TIMER database was used to assess the relationship between LPCAT1 and the degree of infiltration of specific immune cell subpopulations. Furthermore, considering the potential oncogenic role of LPCAT1 in LIHC, the relationship of LPCAT1 with immune checkpoints (including CD274/PDCD1/CTLA4) was also explored [18–21]. To further investigate the potential role of LPCAT1 in tumor immune infiltration, we determined the expression correlation of LPCAT1 with biomarkers of immune cells in LIHC.

### 2.6. Statistical Analysis

The statistical analysis was automatically calculated by the online database mentioned above or via the *R* software. *p* value less than 0.05 was considered statistically significant.

## 3. Result

### 3.1. Analysis of the LPCAT1 Expression in Pan Cancer

**Figure 1:**
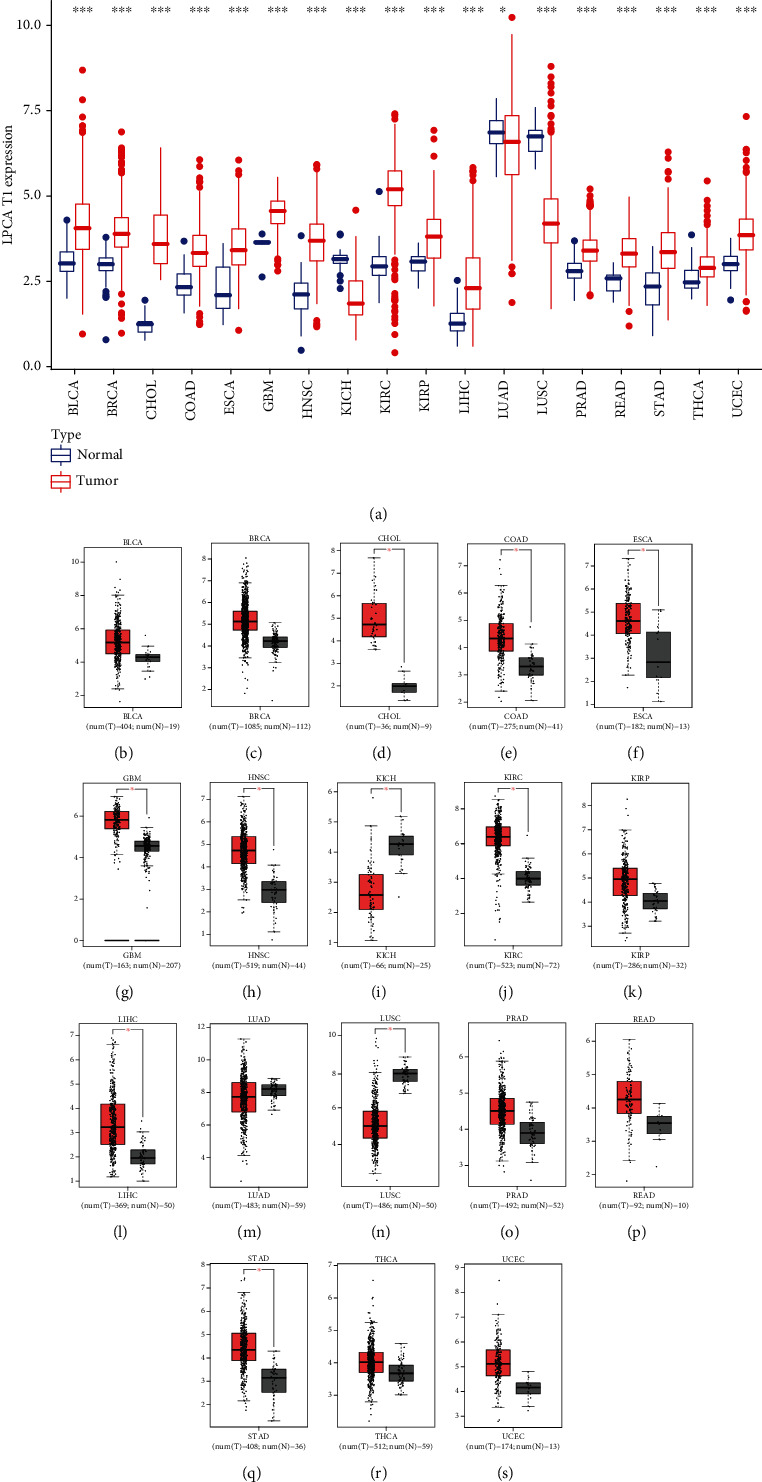
Expression analysis for LPCAT1 in multiple cancers. (a) The expression of LPCAT1 in 18 types of human cancer based on TCGA cancer and normal data. (b)–(m) The LPCAT1 expression in the GEPIA database. BLCA (b), BRCA (c), CHOL (d), COAD (e), ESCA (f), GBM (g), HNSC (h), KICH (i), KIRC (j), KIRP (k), LIHC (l), LUAD (m), LUSC (n), PRAD (o), READ (p), STAD (q), THCA (r), and UCEC (S) tissues compared with corresponding TCGA and GTEx normal tissues. ∗*p* value < 0.05; ∗∗*p* value < 0.01; ∗∗∗*p* value < 0.001.

Firstly, the expression level of LPCAT1 in eighteen types of cancers was evaluated, which found that LPCAT1 was significantly upregulated in 16 cancer types other than KICH and LUSC, compared to corresponding normal samples (Figure 1(a)). Then, we validated the results in the GEPIA database, which revealed that the expression levels of LPCAT1 in CHOL, COAD, ESCA, GBM, HNSC, KIRC, LIHC, and STAD significantly increased, and the expression levels of LPCAT1 in KICH and LUSC significantly decreased, but no significant change in expression level was detected in BLCA, BRCA, KIRP, LUAD, PRAD, READ, THCA, and UCEC (Figure 1(b)).

In combination with the results from the two databases, the LPCAT1 expression was upregulated in CHOL, COAD, ESCA, GBM, HNSC, KIRC, LIHC, and STAD and was downregulated in KICH and LUSC, which indicated that LPCAT1 might be curial in the development of these ten cancers.

### 3.2. The Prognostic Value of LPCAT1 in Human Cancers

**Figure 2:**
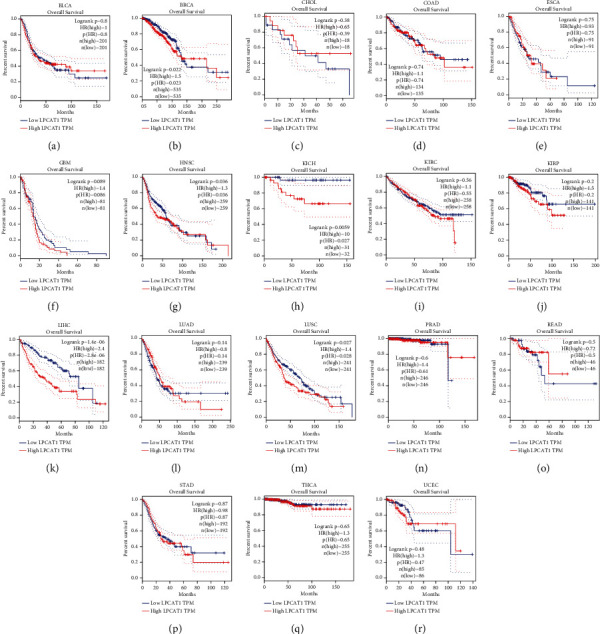
The overall survival (OS) analysis for LPCAT1 in various human cancers determined by the GEPIA database. (a)–(l) The OS plot of LPCAT1 in BLCA (a), BRCA (b), CHOL (c), COAD (d), ESCA (e), GBM (f), HNSC (g), KICH (h), KIRC (i), KIRP (j), LIHC (k), LUAD (l), LUSC (m), PRAD (n), READ (o), STAD (p), THCA (q), and UCEC (r).

**Figure 3:**
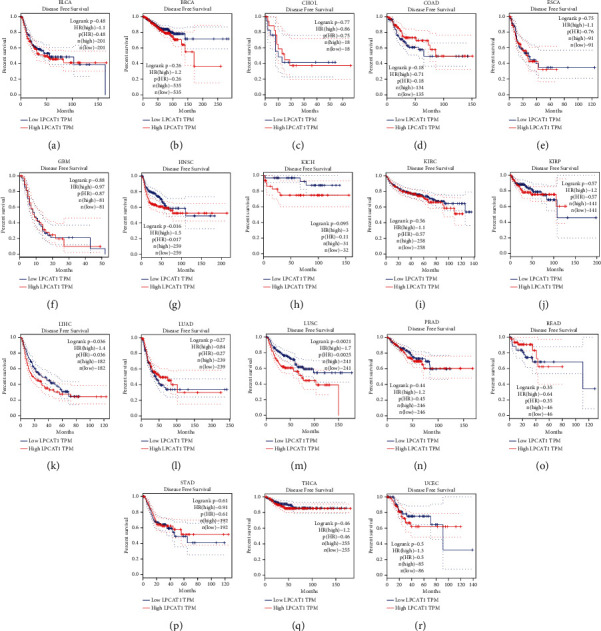
The disease-free survival (RFS) analysis for LPCAT1 in various human cancers determined by the GEPIA database. (a)–(l) The OS plot of LPCAT1 in BLCA (a), BRCA (b), CHOL (c), COAD (d), ESCA (e), GBM (f), HNSC (g), KICH (h), KIRC (i), KIRP (j), LIHC (k), LUAD (l), LUSC (m), PRAD (n), READ (o), STAD (p), THCA (q), and UCEC (r).

Survival analysis in BLCA, BRCA, CHOL, COAD, ESCA, HNSC, KIRC, LIHC, LUAD, LUSC, READ, STAD, and UCEC was performed to explore the prognostic value of LPCAT1. The higher expression of LPCAT1 was associated with worse OS in BRCA, HNSC, KICH, LIHC, and LUSC (Figure 2). In terms of DFS, the high LPCAT1 expression in HNSC, LIHC, and LUSC was associated with poor prognosis (Figure 3). Ultimately, LPCAT1 was found to be a potential biomarker for poor prognosis in patients with HNSC, LIHC, and LUSC.

### 3.3. Prediction and Analysis of Upstream miRNA of LPCAT1

**Figure 4:**
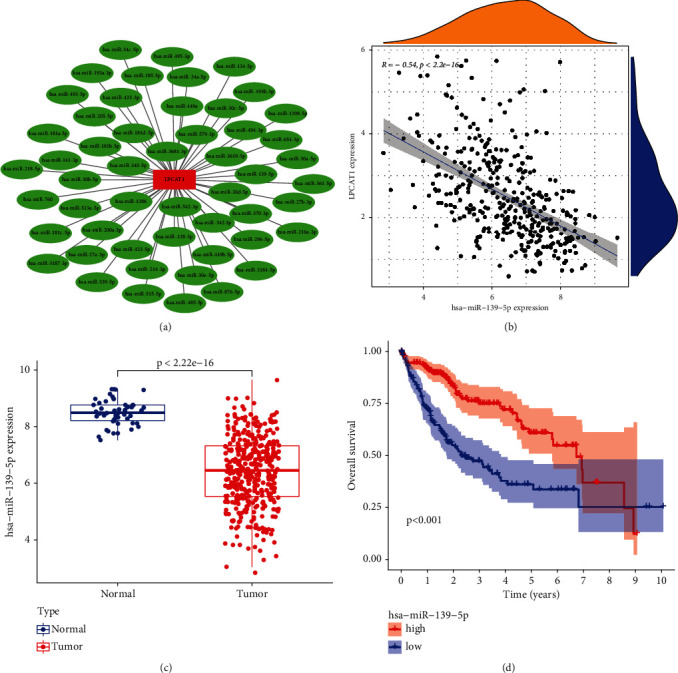
Identification of hsa-miR-195-5p as a potential upstream miRNA of LPCAT1 in LIHC. (a) The miRNA-LPCAT1 regulatory network established by Cytoscape software. (b) The expression correlation between hsa − miR−139−5p and LPCAT1 in LIHC analyzed by *R* software. (c) The expression of hsa − miR−139−5p in LIHC and control normal samples determined by *R* software. (d) The prognostic value of hsa − miR−139−5p in LIHC assessed by *R* software.

The regulation of the gene expression by ncRNA has been widely recognized; so, whether LPCAT1 was modulated by several ncRNAs was urgently ascertained. First, the upstream miRNAs regulating LPCAT1 were predicted by the starBase, which found a total of 52 potential miRNA molecules. Then, the regulatory network of these miRNAs and LPCAT1was established using Cytoscape software (Figure 4(a)). Based on the regulatory relationship between miRNAs and genes, there should be a negative correlation between upstream miRNA and LPCAT1 (*R* < −2, *p* < 0.05). Finally, four miRNA, including hsa-miR-139-5p, hsa-miR-27b-3p, hsa-miR-193b-3p, and hsa-miR-30e-5p, met this condition. Because the correlation coefficient between hsa-miR-139-5p and the target gene was the largest, hsa-miR-139-5p was selected as a qualified miRNA, and its expression and prognostic value in LIHC were next analyzed (Figures 4(b)–4(d)).

### 3.4. Prediction and Analysis of Upstream lncRNAs of hsa-miR-139-5p

**Figure 5:**
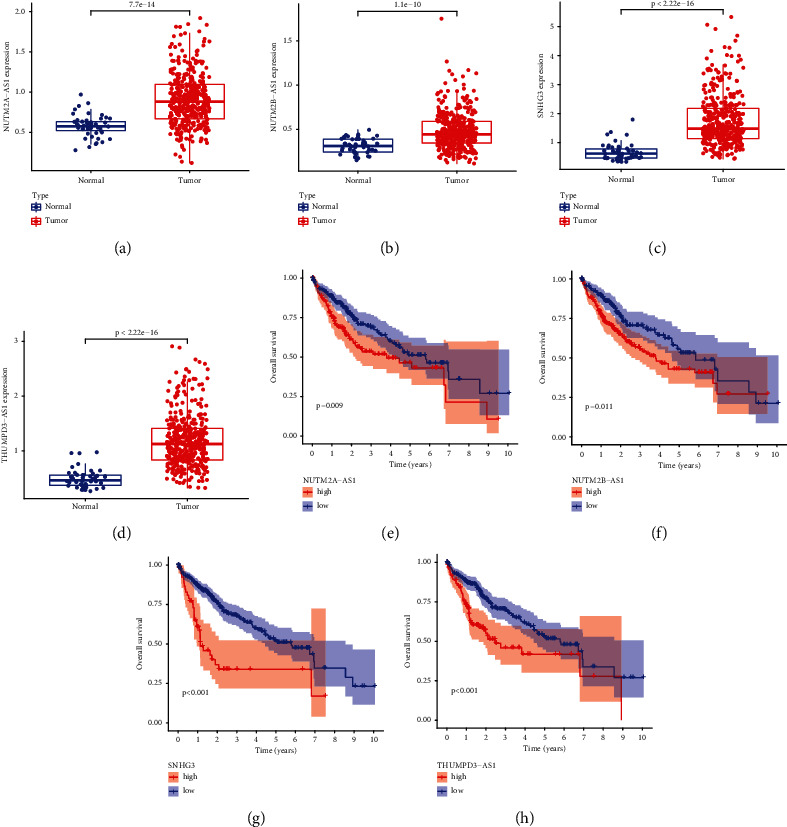
Expression analysis and survival analysis for upstream lncRNAs of hsa − miR−139−5p in LIHC. (a)–(d) The expression of NUTM2A − AS1 (a), NUTM2B − AS1 (b), SNHG3 (c), T HUMPD3 − AS1(d). (f)–(j) The OS analysis for NUTM2A − AS1 (e), NUTM2B − AS1 (f), SNHG3 (g), and T HUMPD3 − AS1 (h) in LIHC.

After finding the upstream miRNA (hsa-miR-139-5p) of LPCAT1, we applied starBase to predict the upstream lncRNA of hsa-miR-139-5p. Similar to the miRNA-LPCAT1 network, lncRNA-hsa-miR-139-5p network was plotted for visualization (Figure S1). As presented in Figure 5, NUTM2A − AS1, NUTM2B − AS1, SNHG3, and THUMPD3 − AS1 were found to be highly expressed in tumor samples. And NUTM2A − AS1, NUTM2B − AS1, SNHG3, and THUMPD3 − AS1 upregulation were negatively correlated with prognosis. According to the ceRNA hypothesis, lncRNA should be negatively correlated with miRNA and positively correlated with mRNA. The coexpression relationship of these lncRNAs with mRNA and miRNA was analyzed. As shown in Table 1, the correlation coefficient between SNHG3 and hsa-miR-139-5p was the largest, and the correlation coefficient between SNHG3 and hsa-LPCAT1 was also the highest. Thus, SNHG3 could be the most promising upstream lncRNA for the miR-139-5p/LPCAT1 axis in LIHC.

### 3.5. LPCAT1 Correlates with Immune Cell Infiltration in LIHC

**Figure 6:**
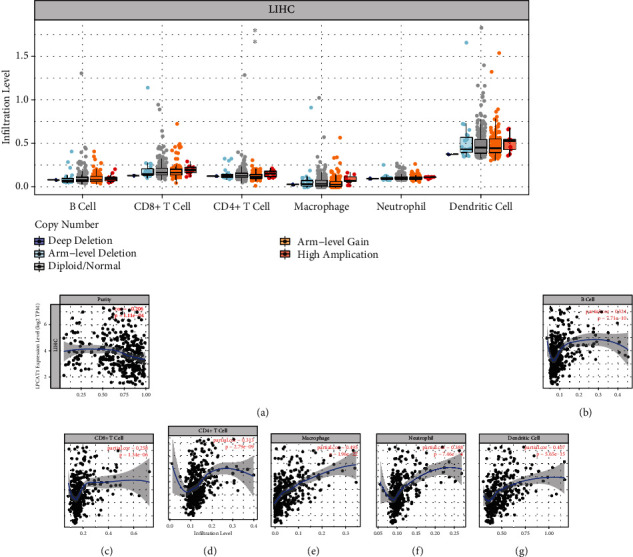
The relationship of immune cell infiltration with LPCAT1 level in LIHC. (a) The infiltration level of various immune cells under different copy numbers of LPCAT1 in LIHC. (b)–(g) The correlation of LPCAT1 expression level with B cell (b), CD8 + T cell (c), CD4 + T cell (d), macrophage (e), neutrophil (f), or dendritic cell (g) infiltration level in LIHC.

Previous studies demonstrated that LPCAT1 played an active role in the immune system and genetics. The level of immune cell infiltration in LIHC did not vary significantly at different LPCAT1 copy numbers except for CD4+ T cell (Figure 6(a)). In addition, we assessed the correlation between LPCAT1 expression levels and immune cell infiltration levels to explore the underlying mechanisms by which LPCAT1 exerts its function. As presented in Figures 6(b)–6(g), the LPCAT1 expression showed a significant positive correlation with B cell, CD8+ T cell, CD4+ T cell, macrophage, neutrophil, and dendritic cell.

### 3.6. Expression Correlation of LPCAT1 and Biomarkers of Immune Cells in LIHC

The role of LPCAT1 in tumor immunity warranted further in-depth study; so, we further explored the correlation between the expression of immune cell biomarkers in LIHC and LPCAT1. The results suggested that LPCAT1 was significantly positively correlated with B-cell biomarkers (CD19 and CD79A), CD8 + T cell's biomarkers (CD8A and CD8B), CD4 + T cell's biomarker (CD4), M1 macrophage biomarkers (NOS2, IRF5, and PTGS2), M2 macrophage biomarkers (CD163, VSIG4, and MS4A4A), neutrophil biomarkers (ITGAM and CCR7), and dendritic cell biomarkers (HLA-DPB1, HLA-DQB1, HLA-DRA, HLA-DPA1, CD1C, ITGAX, and NRP1) (Table 2).

### 3.7. The Relationship between LPCAT1 and Immune Checkpoints in LIHC

**Figure 7:**
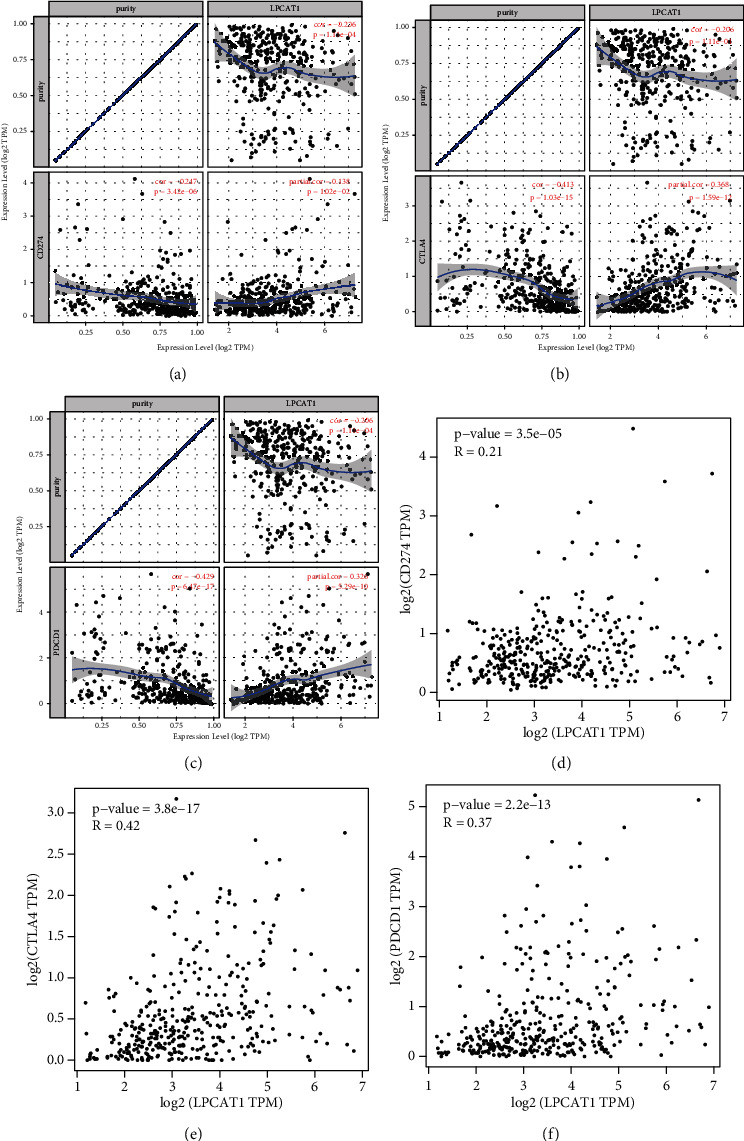
Correlation of the LPCAT1 expression with CD274, PDCD1, and CTLA-4 expression in LIHC. (a) Spearman correlation of LPCAT1 with the expression of CD274 in LIHC adjusted by purity using TIMER. (b) Spearman correlation of LPCAT1 with the expression of PDCD1 in LIHC adjusted by purity using TIMER. (c) Spearman correlation of LPCAT1 with the expression of CTLA-4 in LIHC adjusted by purity using TIMER. (d) The expression correlation of LPCAT1 with CD274 in LIHC determined by the GEPIA database. (e) The expression correlation of LPCAT1 with PDCD1 in LIHC determined by the GEPIA database. (f) The expression correlation of LPCAT1 with CTLA-4 in LIHC determined by the GEPIA database.

As immune checkpoints PD1/PD-L1 and CTLA-4 are critical in the immune escape of tumors, the relationship of LPCAT1 with these immune checkpoints was also assessed. Based on TIMER data analysis, the LPCAT1 expression was significantly positively correlated with PD1, PD-L1, and CTLA-4 in HCC, which was adjusted by purity (Figures 7(a)–7(c)). Similarly, GEPIA data analysis also found significant positive correlation of LPCAT1 with PD1, PD-L1, or CTLA-4 (Figures 7(d)–7(f)).

## 4. Discussion

LIHC is one of the most common gastrointestinal malignancies worldwide, and its main causative factors include alcoholism, drug stimulation, obesity, hepatitis B virus, and hepatitis C virus [1, 22]. The key to the treatment of LIHC is early diagnosis so that the patient can be treated effectively. However, most patients with LIHC are diagnosed at an advanced stage. Therefore, it is important to explore the possible pathogenesis of LIHC and to find molecular markers in the development of LIHC. Previous studies demonstrated that LPCAT1 plays a vital role in different types of human cancer, including LIHC [6, 23–26]. However, the understanding of LPCAT1 in LIHC remains insufficient. Therefore, further investigation of its roles in LPCAT1 is urgently needed.

Firstly, the expression level of LPCAT1 in eighteen types of cancers was evaluated in both TCGA and GEPIA databases, which found that the LPCAT1 expression was upregulated in LIHC. Next, survival analyses for the prognosis of cancer patients further proved the potential role of LPCAT1 in LIHC. In experiments conducted by He et al., they found that increased LPCAT1 correlated with poor prognosis in HCC patients and fueled HCC progression by promoting cellular growth, migration, and metastasis [27]. This study and our results revealed the oncogenic role of LPCAT1 in LIHC.

RNAs in living organisms are diverse and complex in function and are generally classified into two categories according to whether they encode proteins or not: coding RNA (coding RNA) and noncoding RNA (noncoding RNA, ncRNA). The former refers to mRNA, while the latter includes many types, such as the well-known tRNA and rRNA, as well as miRNA and lncRNA, which have been proven to be involved in the regulation of the gene expression [28]. Target gene prediction programs (including TargetScan, miRmap, miRanda, PicTar, RNA22, PITA, microT) in starBase were used to predict the upstream miRNA. Among all 52 possible miRNAs, four miRNAs (hsa-miR-139-5p, hsa-miR-27b-3p, hsa-miR-193b-3p, and hsa-miR-30e-5p) showed significant correlation with LPCAT1. Because the correlation coefficient between hsa-miR-139-5p and the target gene was the largest, hsa-miR-139-5p was selected as a qualified miRNA. The subsequent analysis also showed that hsa-miR-139-5p was lowly expressed in the tumor samples, and lower expression of hsa-miR-139-5p was associated with a poor prognosis of LIHC. During the past decade, several studies have investigated the role of hsa-miR-139-5p in cancers, including ovarian cancer, thyroid cancer, lung cancer, and LIHC [29–31]. Tu et al. found that nuclear enriched abundant transcript 1 (NEAT1) can upregulate TGF-*β*1 to induce hepatocellular carcinoma progression by sponging hsa-mir-139-5p [32]. The above experimental findings are consistent with our results. In conclusion, hsa-mir-139-5p was finally found to be an important regulatory molecule of LPCAT1 in LIHC.

According to the ceRNA hypothesis, ceRNA can bind to miRNA and thus inhibit the effect of miRNA on mRNA. Therefore, the potential lncRNA of the hsa-mir-139-5p/LPCAT1 axis was explored as it could be potentially oncogenic. Finally, NUTM2A − AS1, NUTM2B − AS1, SNHG3, and THUMPD3 − AS1 were identified to be highly expressed in tumor samples and were negatively correlated with prognosis. The most potential upregulated lncRNA, SNHG3, was selected, as it was found to be the most negatively associated with hsa-miR-125b-5p and most positively correlated with LPCAT1. As reported by Xie et al., SNHG3 functions in an oncogenic manner to drive gastric cancer proliferation, migration, and invasion by regulating the miR-139-5p/MYB axis [33]. Zhao et al. proposed that SNHG3 promotes LIHC progression via the miR-326/SMAD3/ZEB1 signaling pathway [34]. Combined with the present study and previous findings, SNHG3-miR-139-5p-LPCAT1 may be a potential regulatory pathway in LIHC.

In recent years, tumor immunotherapy has played an increasingly important role, and more studies tend to explore the mechanisms of tumor microenvironment regulation of immune cell function. For example, immune cell infiltration is closely related to tumorigenesis and prognosis and deserves further study [35]. In our research, the results showed that there was a positive correlation between LPCAT1 expression and neutrophil, macrophage, dendritic cell, CD4 + T cell, CD8 + T cell, and B cell in LIHC. Additionally, the LPCAT1 expression was also associated with biomarkers of infiltrating immune cells, including CD19 and CD79A (B-cell biomarkers), CD8A and CD8B (CD8 + T cell's biomarkers), CD4 (CD4 + T cell's biomarker), NOS2, IRF5, and PTGS2 (M1 macrophage biomarkers), CD163, VSIG4, and MS4A4A (M2 macrophage biomarkers), ITGAM and CCR7 (neutrophil biomarkers), and HLA-DPB1, HLA-DQB1, HLA-DRA, HLA-DPA1, CD1C, ITGAX, and NRP1 (dendritic cell biomarkers). The results also suggested that high expression of LPCAT1 was strongly linked to CD274, PDCD1, or CTLA-4 in HCC, indicating that targeting LPCAT1 might increase the efficacy of immunotherapy in LIHC. These results suggest that tumor immune infiltration could play a vital role in LPCAT1-mediated development of LIHC.

**Figure 8:**
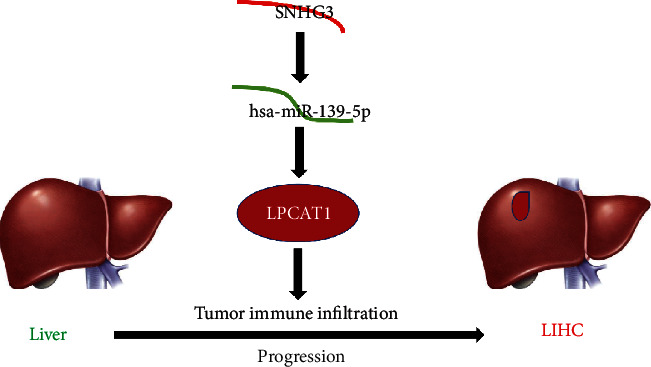
The model of the SNHG3-hsa-miR-195-5p-LPCAT1 axis in carcinogenesis of LIHC.

In conclusion, SNHG3 was selected as a possible upstream lncRNA for miR-139-5p, thereby affecting the LPCAT1 expression and promoting hepatocellular carcinoma progression. Our study suggests that SNHG3 may be a potential therapeutic target and prognostic indicator. Besides, LPCAT1 may play its tumorigenic role by enhancing tumor immune cell infiltration and immune checkpoint expression (Figure 8). However, these findings still need to be further proved with more benchwork studies and clinical studies.

## Figures and Tables

**Table 1:** Correlation analysis between lncRNA and hsa-miR-139-5p or LPCAT1 in LIHC determined by the starBase database.

lncRNA	Gene/miRNA	Cor	*p* value
NUTM2A-AS1	LPCAT1	0.311045764	1.19*E*-09
THUMPD3-AS1	LPCAT1	0.397802325	6.87*E*-16
SNHG3	LPCAT1	0.495761543	0
NUTM2B-AS1	LPCAT1	0.363806539	6.97*E*-13
NUTM2A-AS1	hsa-miR-139-5p	-0.249437433	1.28*E*-06
THUMPD3-AS1	hsa-miR-139-5p	-0.383988472	2.16*E*-14
SNHG3	hsa-miR-139-5p	-0.427905007	0
NUTM2B-AS1	hsa-miR-139-5p	-0.291498033	1.30*E*-08

**Table 2:** Correlation analysis between LPCAT1 and biomarkers of immune cells in LIHC.

Immune cell	Gene	Cor	*p* value
B cell	CD19	0.2965	5.01*E*-09
B cell	CD79A	0.18487	0.000325
CD8+ T cell	CD8A	0.229626	7.69*E*-06
CD8+ T cell	CD8B	0.231026	6.36*E*-06
CD4+ T cell	CD4	0.203542	7.60*E*-05
M1 macrophage	NOS2	-0.12516	0.015441
M1 macrophage	IRF5	0.258382	4.46*E*-07
M1 macrophage	PTGS2	0.243057	1.97*E*-06
M2 macrophage	CD163	0.185346	0.000321
M2 macrophage	VSIG4	0.279263	4.53*E*-08
M2 macrophage	MS4A4A	0.269175	1.40*E*-07
Neutrophil	CEACAM8	0.076488	0.13983
Neutrophil	ITGAM	0.438427	0
Neutrophil	CCR7	0.107446	0.037846
Dendritic cell	HLA-DPB1	0.329099	8.77*E*-11
Dendritic cell	HLA-DQB1	0.284063	2.61*E*-08
Dendritic cell	HLA-DRA	0.303176	2.61*E*-09
Dendritic cell	HLA-DPA1	0.294587	7.50*E*-09
Dendritic cell	CD1C	0.080788	0.118835
Dendritic cell	NRP1	0.279079	4.63*E*-08
Dendritic cell	ITGAX	0.424075	0

## Data Availability

Publicly available datasets were analyzed in this study. This data can be found here: TCGA database: https://portal.gdc.cancer.gov/. Further inquiries can be directed to the corresponding author.

## Abbreviations

LIHC: Liver hepatocellular carcinoma
ncRNAs: Nonencoding RNAs
TCGA: The Cancer Genome Atlas
TIMER: Tumor Immune Estimation Resource
LPCATs: Lysophosphatidylcholine acyltransferases
LPC: Lysophosphatidylcholine
PC: Phosphatidylcholine
miRNAs: MicroRNAs
lncRNAs: Long noncoding RNAs
GTEx: Genotype-tissue expression.

## Supplementary Materials

Supplementary Materials Supplementary Figure 1: the LncRNA hsa-miR-195-5p regulatory network established by Cytoscape software.
