# Supplementary figures and images for: ncRNA-Mediated High Expression of LPCAT1 Correlates with Poor Prognosis and Tumor Immune Infiltration of Liver Hepatocellular Carcinoma
Source: J Immunol Res. 2022 May 16;2022:1584397. doi: 10.1155/2022/1584397 (PMC9126685; doi:10.1155/2022/1584397)

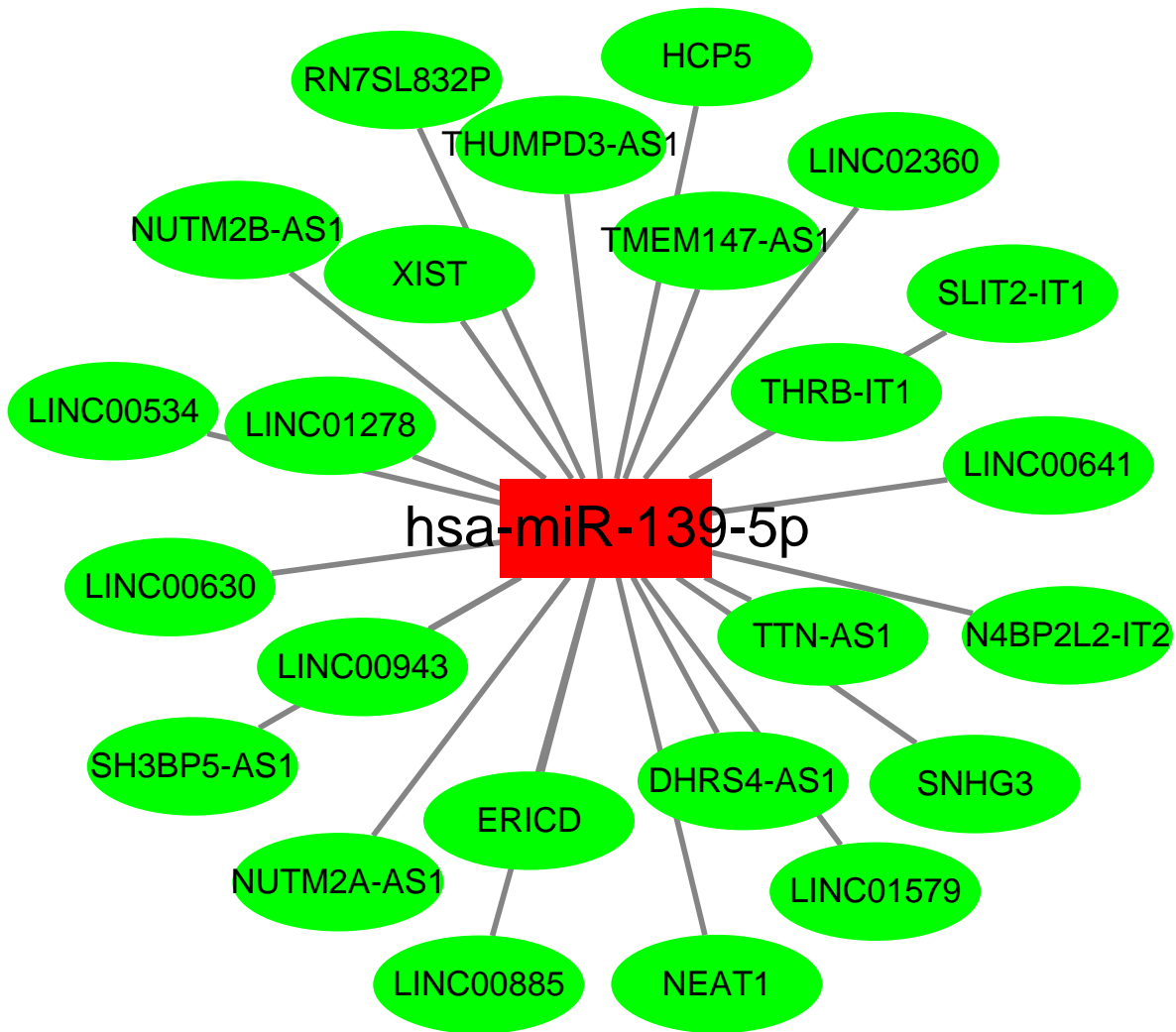

Supplement: Supplementary Materials — Supplementary Figure 1: the LncRNA hsa-miR-195-5p regulatory network established by Cytoscape software. [file 1584397.f1.pdf]
